# Few-shot learning for the classification of intestinal tuberculosis and Crohn's disease on endoscopic images: A novel learn-to-learn framework

**DOI:** 10.1016/j.heliyon.2024.e26559

**Published:** 2024-02-16

**Authors:** Jiaxi Lin, Shiqi Zhu, Minyue Yin, Hongchen Xue, Lu Liu, Xiaolin Liu, Lihe Liu, Chunfang Xu, Jinzhou Zhu

**Affiliations:** a Department of Gastroenterology, The First Affiliated Hospital of Soochow University, Suzhou, Jiangsu, 215006, China; b Suzhou Clinical Centre of Digestive Diseases, Suzhou, Jiangsu, 215006, China; c School of Computer Science and Technology, Soochow University, Suzhou, Jiangsu, 215006, China

**Keywords:** Few-shot learning, Intestinal tuberculosis, Crohn's disease, Meta learning

## Abstract

**Background and aim:**

Standard deep learning methods have been found inadequate in distinguishing between intestinal tuberculosis (ITB) and Crohn's disease (CD), a shortcoming largely attributed to the scarcity of available samples. In light of this limitation, our objective is to develop an innovative few-shot learning (FSL) system, specifically tailored for the efficient categorization and differential diagnosis of CD and ITB, using endoscopic image data with minimal sample requirements.

**Methods:**

A total of 122 white-light endoscopic images (99 CD images and 23 ITB images) were collected (one ileum image from each patient). A 2-way, 3-shot FSL model that integrated dual transfer learning and metric learning strategies was devised. Xception architecture was selected as the foundation and then underwent a dual transfer process utilizing oesophagitis images sourced from HyperKvasir. Subsequently, the eigenvectors derived from the Xception for each query image were converted into predictive scores, which were calculated using the Euclidean distances to six reference images from the support sets.

**Results:**

The FSL model, which leverages dual transfer learning, exhibited enhanced performance metrics (AUC 0.81) compared to a model relying on single transfer learning (AUC 0.56) across three evaluation rounds. Additionally, its performance surpassed that of a less experienced endoscopist (AUC 0.56) and even a more seasoned specialist (AUC 0.61).

**Conclusions:**

The FSL model we have developed demonstrates efficacy in distinguishing between CD and ITB using a limited dataset of endoscopic imagery. FSL holds value for enhancing the diagnostic capabilities of rare conditions.

## 1 Introduction

Due to recent advances in machine learning, deep learning (DL) has gained widespread adoption across numerous clinical applications within the domain of endoscopy, including identifying early gastrointestinal cancers, detecting colorectal polyps, performing standardized assessments of inflammatory bowel disease, *etc.* [[1], [2], [3]]. Relying on high-quality endoscopic image databases, DL has demonstrated excellence regarding the management of common digestive diseases [4]. However, reports concerning the application of DL for uncommon diseases are limited. The reason for this is that standard DL performs poorly on small samples due to the underfitting problem that occurs during the training process [5]. Intestinal tuberculosis (ITB) and Crohn's disease (CD) are uncommon digestive diseases, especially the former. The similarities between the endoscopic manifestations of CD and ITB make their diagnosis a challenge for endoscopists, especially for undertrained endoscopists [6,7]. Concurrently, the limited availability of endoscopic imagers further exacerbates the challenges posed to the effective implementation of DL.

Recently, few-shot learning (FSL) has been propose; it offers a potential solution for training stable and discriminative models from limited samples using metric learning [8]. Our objective in this research was to construct a 2-way, 3-shot FSL framework to distinguish between rare conditions, such as CD and ITB, using endoscopic images.

## 2 Methods

### 2.1 Study populations and image selection

A retrospective study was conducted, including individuals diagnosed with either CD or ITB between 2014 and 2020. The diagnosis of ITB was based on one of the following criteria: 1) pathological confirmation: the presence of caseating granuloma in intestinal biopsies, surgically removed tissue, or mesenteric lymph nodes; 2) clinical diagnosis: achieving complete clinical remission with endoscopic evidence of mucosal healing following a minimum of six months of standard antituberculosis treatment, and maintaining this remission without relapse during a 9–12 month post-treatment follow-up. The diagnosis of CD was based on one of the following criteria: 1) pathologic diagnosis: the histological examination of tissue samples confirmed CD, with the absence of caseating granuloma in the intestine or mesenteric lymph nodes; 2) clinical diagnosis: a favourable response to CD therapy has been observed based on clinical signs, laboratory tset, and endoscopy or radiology, and a consistent disease trajectory over a minimum of one year, including endoscopic mucosal healing [9].

We collected one white-light endoscopic image of ileum lesions from each eligible patient. The images were selected by two senior endoscopists (with more than 10 years of experience). The research received ethical approval from the Institutional Review Board at the First Affiliated Hospital of Soochow University, with the assigned approval number 2022098.

### 2.2 FSL model development

We adopted metric learning based on dual transfer learning to design and develop the model. The detailed procedure was executed as follows.

**1) Network structure selection.** Given the small sample size, the selection of an appropriate feature extraction network structure was important. The high-performance Xception network, which integrates residual connections, global average pooling, and a depthwise separable convolution module, was adopted for feature extraction [10].

2) **Transfer learning**. Transfer learning is a pivotal methodology in the domain of deep learning that facilitates knowledge transfer between different, yet related, tasks. It deviates from the conventional approach of training models de novo for every new problem by capitalizing on the pre-existing learned features from one task to enhance performance and reduce data dependency in another.

Prior knowledge provided by transfer learning can enhance the learning efficiency and prediction ability of FSL. ImageNet and HyperKvasir have been proven successful for transfer learning. ImageNet, which contains 14,197,122 annotated images according to the WordNet hierarchy, was first used to provide the FSL model with generalization experience [11]. HyperKvasir is a comprehensive multiclass image database for gastrointestinal endoscopy that provides model-specific FSL knowledge concerning digestive endoscopy [12]. Xception was first pretrained on ImageNet and subsequently underwent a dual-pretrained phase, focusing on a binary classification task that utilized oesophagitis images (883 normal zline images vs. 269 images of oesophagitis classes B/C/D, based on the Los Angeles Classification [13]) from HyperKvasir.

**3) Metric learning.** The metric learning process involved two support sets with two classes (10.13039/100011639CD and ITB) and a query set of endoscopic images to be classified. We set the framework as a 2-way, 3-shot structure in a random three-round manner (2-way means two classifications; 3-shot means that three images were present in each support set). The Euclidean distance measure served as the classifier. The Euclidean distance of the eigenvector (a 1*50 feature vector extracted by Xception for each image) between each query and six support set images (three images * two classifications) determined the model predictions. The Euclidean distance from one query image to one support set image was calculated as shown in Equation (1):

(1) $$D=\sqrt{{\left({q_{1}-s}_{1}\right)}^{2}+{\left({q_{2}-s}_{2}\right)}^{2}\cdots +{\left({q_{50}-s}_{50}\right)}^{2}}=\sqrt{\sum_{i=1}^{50}{\left(q_{i}-s_{i}\right)}^{2}}$$

D, Euclidean distance; q, query set; s, support set; 50, 1*50 eigenvector.

**Fig. 1:**
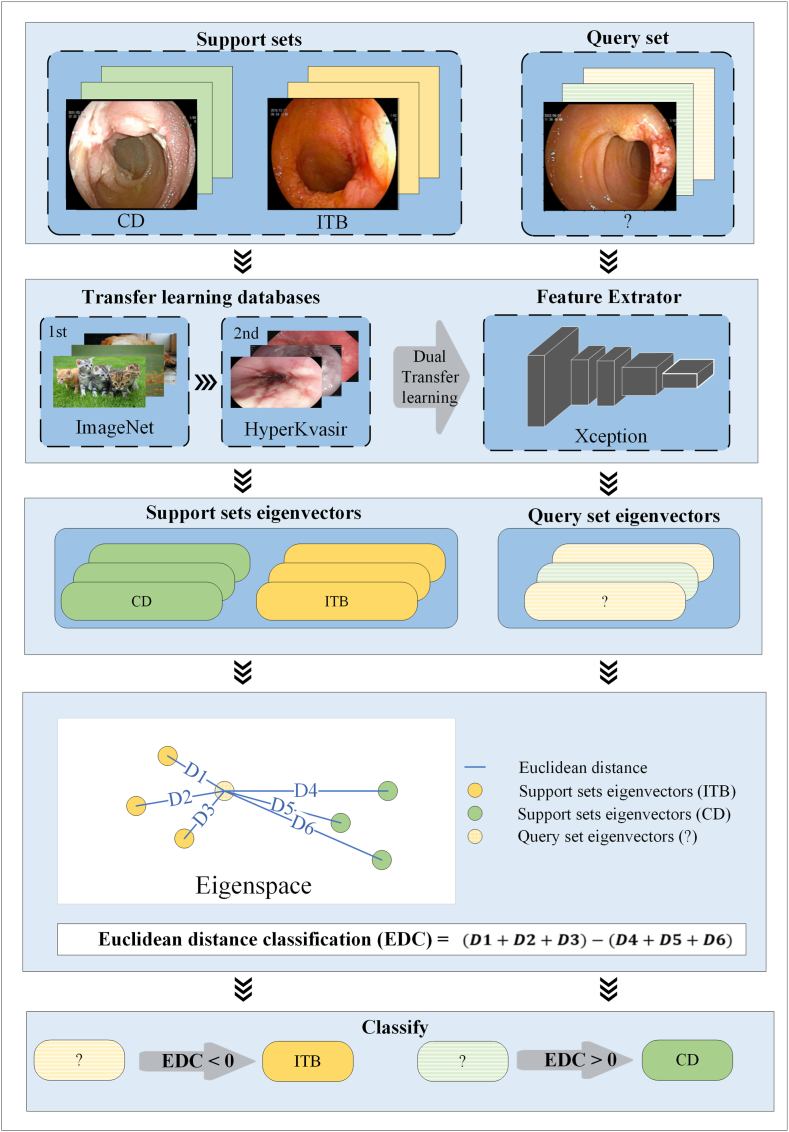
**The framework of the few-shot learning model.** CD: Crohn's disease. ITB: intestinal tuberculosis. EDC：Euclidean distance classification.

The study was conducted in three rounds to ensure the reliability of the results. The framework of the FSL model is shown in Fig. 1.

### 2.3 Few-shot learning model evaluation and interpretation

The classification task was conducted by a junior endoscopist, who had fewer than five years of endoscopic practice, and a senior endoscopist, with over a decade of experience, to facilitate comparative analysis. The performance of the model was assessed using a range of metrics such as the area under the curve (AUC), accuracy, sensitivity, specificity, recall, precision, and F1-score.

The gradient-weighted class activation mapping (Grad-CAM) algorithm was used to visually interpret the model [14]. Grad-CAM uses the gradients of the last fully connected layer and flows into the final convolutional layer to make a localization map highlighting the significant areas in the images. The results of Grad-CAM are presented in the form of heatmaps.

### 2.4 Software

Keras (backbone: TensorFlow Version:2.8.0) based on Python (Version 3.8.0) was used to train the Xception model and obtain eigenvectors. R (Version 4.3.0) was used for data analysis.

## 3 Results

### 3.1 Study population

A total of 23 ITB confirmed patients and 99 CD patients were included in this retrospective study. The characteristics of basic information, endoscopy and pathology were listed in Table 1.

**Table 1:** Characteristics of patients.

	Variable	ITB (n = 23)	CD (n = 99)	*P* value
**Demographic profile**	Age (yrs)	33.7 ± 11.9	29.9 ± 13.4	0.214
	Sex (Male)	14 (60.7%)	46 (46.5%)	0.213
	Body mass index	21.8 ± 4.6	20.7 ± 5.2	0.353
**Symptom**	Abdominal pain	17 (73.9%)	78 (78.8%)	0.612
	Diarrhea	8 (34.8%)	61 (61.6%)	0.019
	Gastrointestinal bleeding	11 (47.8%)	48 (48.5%)	0.955
	Fever	5 (21.7%)	8 (8.1%)	0.056
	Weight loss	17 (73.9%)	63 (63.6%)	0.350
	Perianal lesion	2 (8.7%)	33 (33.3%)	0.021
	Anal fistula	2 (8.7%)	37 (37.4%)	0.007
	Intestinal obstruction	5 (21.7%)	11 (11.1%)	0.174
	Extra-intestinal manifestations	6 (26.1%)	46 (46.5%)	0.075
	Extra-intestinal tuberculosis	12 (52.2%)	0 (0.0%)	<0.001
**Laboratory test**	Stool OB test (positive)	15 (65.2%)	57 (57.6%)	<0.001
	T-spot (positive)	14 (60.9%)	2 (2.02%)	<0.001
	Albumin (g/L)	32.6 ± 5.7	30.8 ± 66	0.230
	CRP (mg/L)	24.4 ± 13.2	36.0 ± 14.1	<0.001
	ESR (mm/h)	41.2 ± 17.4	32.8 ± 15.9	0.027
**Colonoscopic findings**	Longitudinal ulcers	2 (8.77%)	51 (51.5%)	<0.001
	Cobble stone appearance	3 (13.0%)	57 (57.6%)	<0.001
	Aphthous ulcers	4 (17.4%)	39 (39.4%)	0.055
	Pseudopolyps	10 (43.4%)	44 (44.4%)	0.933
	Scars	3 (13.0%)	23 (23.2%)	0.400
**Pathological findings**	Granulomas	18 (78.3%)	69 (69.7%)	0.609
	Caseous necrosis	7 (30.4%)	0 (0.0%)	<0.001
	Non-caseous necrosis	17 (73.9%)	52 (52.5%)	0.062
	Acid fast bacilli staining (positive)	5 (21.7%)	0 (0.0%)	<0.001
	Granulomas	18 (78.3%)	69 (69.7%)	0.609

Abbreviation: ESR, erythrocyte sedimentation rate; CRP, high sensitivity C-reactive protein; OB, occult blood.

### 3.2 Model evaluation

Consequently, the study compiled a dataset comprising 99 images indicative of CD and 23 images depicting ITB. A summary of the FSL models' performance can be found in Table 2. The performance metrics for the FSL model, which incorporates dual transfer learning, include a mean accuracy of 0.78, sensitivity of 0.73, specificity at 0.79, recall rate of 0.73, precision at 0.43, F1-score of 0.54, and an AUC value of 0.81, were higher than those of the FSL model with single transfer learning.

**Table 2:** The performance of few-shot learning models based on single and dual transfer learning.

Model	Accuracy	Sensitivity	Specificity	Recall	Precision	F1-score	AUC
Round #1							
Single	0.65	0.57	0.66	0.57	0.27	0.36	0.59
Dual	**0.81**	**0.81**	**0.81**	**0.81**	**0.48**	**0.61**	**0.83**
Round #2							
Single	0.59	0.42	0.63	0.42	0.21	0.27	0.51
Dual	**0.72**	**0.71**	**0.72**	**0.71**	**0.36**	**0.48**	**0.78**
Round #3							
Single	0.64	0.38	0.69	0.38	0.21	0.27	0.59
Dual	**0.80**	**0.66**	**0.83**	**0.66**	**0.46**	**0.54**	**0.84**

Abbreviation: AUC: area under the curve. Single: single transfer learning. Dual: dual transfer learning.

**Fig. 2:**
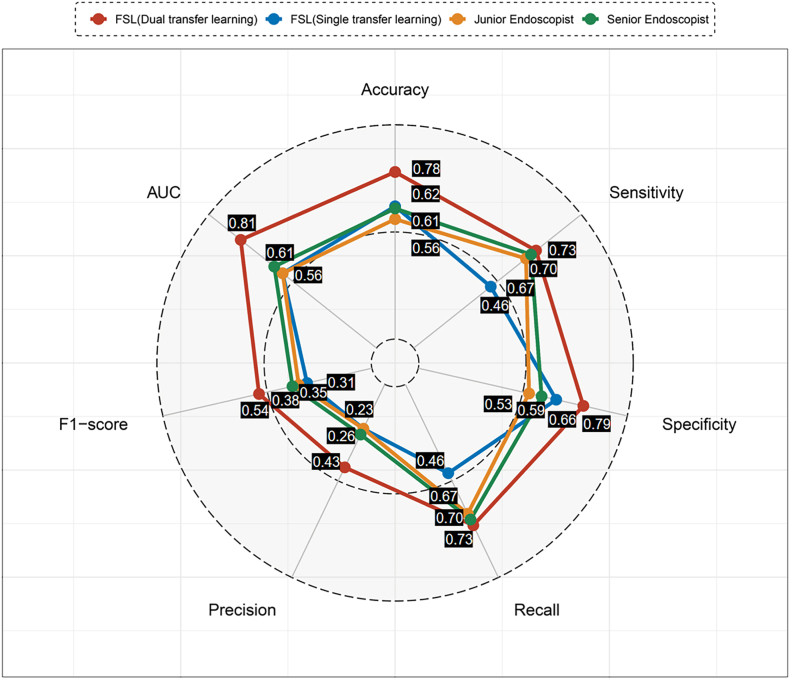
**The performance of few-shot learning models and endoscopists.** AUC: area under the curve.

The comparative performance of the endoscopists and the average performance achieved by FSL models across three evaluation rounds is depicted in Fig. 2. The FSL model utilizing dual transfer learning outperformed both the senior endoscopist, with metrics of accuracy 0.61 and AUC 0.61, and the junior endoscopist, with metrics of accuracy 0.56 and AUC 0.56.

### 3.3 Model interpretation

**Fig. 3:**
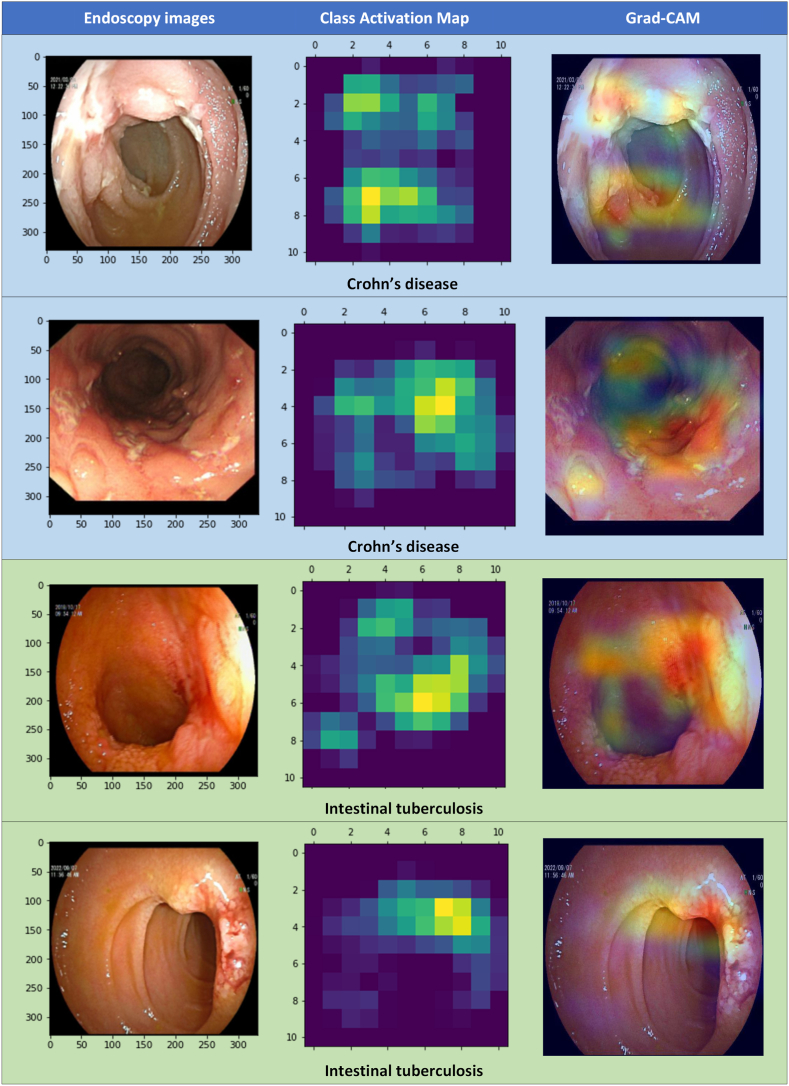
**The visual interpretation of the few-shot learning model.** Grad-CAM: Gradient-weighted Class Activation Mapping.

The Grad-CAM plots are shown in Fig. 3. Four endoscopic images, consisting of 2 CD images and 2 ITB images, were chosen for visual interpretation examples. Grad-CAM was based on the overlay of raw endoscopic images and class activation maps. The highlighted regions in the Grad-CAM plots were the lesions considered by the FSL model.

## 4 Discussion

The research involved the development of FSL models that integrates oesophagitis-based dual transfer learning and Euclidean distance metric learning for the differentiation of CD and ITB using ileum endoscopic images. Upon multiple-round evaluations, the proposed dual transfer learning FSL model displayed superior discriminatory capabilities and consistently robust performance compared to both individual endoscopists and models utilizing single transfer learning.

FSL, an extension and complement to conventional DL methods, offers a distinct advantage by necessitating fewer samples while demonstrating rapid adaptability to novel tasks not included in the training data. FSL is useful for model training cases in which limited data are available, especially for the recognition and classification of uncommon diseases in various medical datasets. In a previous study, Suganya D et al. proposed an FSL model based on a residual network with 50 layers to predict the severity levels of lung disorders induced by COVID-19 [15]. Liu et al. developed a diagnostic model for arrhythmia with few abnormal ECG samples using metric meta-learning [16]. However, to the best of our knowledge, reports regarding FSL are rare in the field of endoscopy. To date, only Khadka et al. have proposed a novel approach for the segmentation of colorectal polyp images using implicit model-agnostic meta-learning, which is another kind of FSL strategy [17]. Our research represents an effort in creating an FSL model that employs metric learning for the classification of rare digestive disease.

A few previous studies concerning the classification of CD and ITB have been conducted. June et al. developed a seven-variable (age, diarrhea, ring-shaped ulcers, longitudinal ulcers, sigmoid involvement, suspicious radiological pulmonary tuberculosis, and gender) classification model that achieved an AUC of 0.97 [18]. He et al. developed a diagnostic nomogram with an AUC of 0.97 by adding special laboratory results (purified protein derivative skin tests and interferon-g release assays) and computed tomography enterorrhaphy features (comb signs) [19]. Previous studies were all based on the fusion of multimodal medical information, which is not suitable for patients with initial endoscopy examinations and limited samples. Moreover, these studies typically relied on endoscopic and radiological parameters that necessitate expert interpretation, thereby inherently being influenced by the subjective experience of the professionals involved. In our study, the transfer learning process of the FSL model only relied on the dual pretraining of deep convolutional neural networks. Furthermore, the outputs of metric learning were calculated with the Euclidean distances determined by eigenvectors. Thus, the classification procedure did not require the participation of physicians in the whole framework.

This study has several limitations. First, the retrospective nature of the study led to potential selection bias. More external test sets based on a prospective design are necessary to assess the generalizability of the developed model. Second, we only evaluated the performance of the FSL model on a binary classification task, and in the future, a multicentral investigation concerning multiclassification is needed.

In summary, we have successfully developed a two-way, three-shot FSL model for the differentiation of CD and ITB using ileum endoscopic images. The FSL model, which leverages dual transfer learning, has exhibited superior performance in a three-round evaluation, surpassing the capabilities of both junior and senior endoscopists, as well as a model based on single transfer learning. Given the constraints of limited data, the FSL approach holds significant potential for enhancing the computer-assisted diagnosis of rare diseases.

## Funding

This work was supported by the 10.13039/501100001809National Natural Science Foundation of China [grant number: 82000540], Suzhou Clinical Centre of Digestive Diseases [grant number: Szlcyxzx202101], Youth Program of Suzhou Health Committee [grant number: KJXW2019001] and Science and Technology Plan of Suzhou City [grant number: SKY2021038].

## Ethical considerations

This retrospective study was approved by the ethics committee of the First Affiliated Hospital of Soochow University (Approval number 2022098).

## Data availability statement

All the code used to extract features, generate models and evaluate model performance can be found in an open-accessed website (https://osf.io/7aujc).

## CRediT authorship contribution statement

**Jiaxi Lin:** Writing – original draft, Investigation, Formal analysis. **Shiqi Zhu:** Data curation. **Minyue Yin:** Data curation. **Hongchen Xue:** Formal analysis. **Lu Liu:** Data curation. **Xiaolin Liu:** Data curation. **Lihe Liu:** Data curation. **Chunfang Xu:** Supervision, Conceptualization. **Jinzhou Zhu:** Writing – review & editing, Supervision, Funding acquisition, Conceptualization.

## Declaration of competing interest

The authors declare that they have no known competing financial interests or personal relationships that could have appeared to influence the work reported in this paper.

## References

[1] Chahal D., Byrne M.F. (2020). A primer on artificial intelligence and its application to endoscopy. Gastrointest. Endosc..

[2] Sharma P., Hassan C. (2022). Artificial intelligence and deep learning for upper gastrointestinal neoplasia. Gastroenterology.

[3] Lo B., Liu Z., Bendtsen F., Igel C., Vind I., Burisch J. (2022). High accuracy in classifying endoscopic severity in ulcerative colitis using convolutional neural network. Am. J. Gastroenterol..

[4] Houwen B.B.S.L., Nass K.J., Vleugels J.L.A., Fockens P., Hazewinkel Y., Dekker E. (2023). Comprehensive review of publicly available colonoscopic imaging databases for artificial intelligence research: availability, accessibility, and usability. Gastrointest. Endosc..

[5] Lei S., Zhang H., Wang K., Su Z. (2022). How training data affect the accuracy and robustness of neural networks for image classification. https://openreview.net/forum?id=HklKWhC5F7.

[6] Kedia S., Das P., Madhusudhan K.S. (2019). Differentiating Crohn's disease from intestinal tuberculosis. World J. Gastroenterol..

[7] Lee Y.J., Yang S.-K., Byeon J.-S. (2006 Jun). Analysis of colonoscopic findings in the differential diagnosis between intestinal tuberculosis and Crohn's disease. Endoscopy.

[8] Snell J., Swersky K., Zemel R.S. (2017).

[9] Ouyang Q., Tandon R., Goh K.L., Pan G.-Z., Fock K.M., Fiocchi C. (2006). Management consensus of inflammatory bowel disease for the Asia-Pacific region. J. Gastroenterol. Hepatol..

[10] Chollet F. (2017).

[11] Russakovsky O., Deng J., Su H. (2015).

[12] Borgli H., Thambawita V., Smedsrud P.H. (2020 Aug). HyperKvasir, a comprehensive multi-class image and video dataset for gastrointestinal endoscopy. Sci. Data.

[13] Lundell L.R., Dent J., Bennett J.R. (1999). Endoscopic assessment of oesophagitis: clinical and functional correlates and further validation of the Los Angeles classification. Gut.

[14] Automated Multimodal Machine Learning for Esophageal Variceal Bleeding Prediction Based on Endoscopy and Structured Data | SpringerLink [Internet]. [cited 2023 Mar 8]. Available from: https://link.springer.com/article/10.1007/s10278-022-00724-6. 10.1007/s10278-022-00724-6 PMC9984604 36279027

[15] D S R.K. (2023). Prognosticating various acute covid lung disorders from COVID-19 patient using chest CT Images. Eng. Appl. Artif. Intell..

[16] Liu Z., Chen Y., Zhang Y., Ran S., Cheng C., Yang G. (2023). Diagnosis of arrhythmias with few abnormal ECG samples using metric-based meta learning. Comput. Biol. Med..

[17] Khadka R., Jha D., Hicks S. (2022). Meta-learning with implicit gradients in a few-shot setting for medical image segmentation. Comput. Biol. Med..

[18] Jung Y., Hwangbo Y., Yoon S.M. (2016). Predictive factors for differentiating between crohn's disease and intestinal tuberculosis in Koreans. Am. J. Gastroenterol..

[19] Y H Z.Z., Y C. (2019). Development and validation of a novel diagnostic nomogram to differentiate between intestinal tuberculosis and crohn's disease: a 6-year prospective multicenter study. Am. J. Gastroenterol..

